# Atypical lipomatous tumor/well-differentiated liposarcoma of the breast: A rare case report

**DOI:** 10.1097/MD.0000000000044108

**Published:** 2025-08-29

**Authors:** Xinrong Qu, Chen Li, Chun Zhang

**Affiliations:** a Department of Breast Surgery, Peking University International Hospital, Beijing, China.

**Keywords:** atypical lipomatous tumor/well-differentiated liposarcoma, breast liposarcoma, prognosis, treatment

## Abstract

**Rationale:**

Liposarcoma, the most common type of soft tissue sarcoma, typically occurs in the deep soft tissues of the limbs and retroperitoneum and is rarely found in the breast. The fifth edition of the World Health Organization classifies it into 5 distinct histological subtypes. Given the rarity of liposarcomas in breast tissue, there remain certain challenges in diagnosis and treatment planning. In this report, we present a rare case of an atypical lipomatous tumor/well-differentiated liposarcoma in breast tissue.

**Patient concerns:**

A 65-year-old female patient with an asymptomatic movable mass in the left breast was admitted to our hospital. The mass persisted for approximately half a month. Clinical examination and ultrasound examination revealed that the mass within the fatty layer of the left breast was approximately 7.0 cm × 7.0 cm and relatively well circumscribed.

**Diagnoses:**

The preoperative tests led to a preliminary diagnosis of lipoma; therefore, the patient underwent mass resection of the left breast under local anesthesia. Postoperative pathological analysis indicated a diagnosis of atypical lipomatous tumor/well-differentiated liposarcoma.

**Interventions:**

Considering the lower malignancy of this pathological type, we performed radical surgical excision and followed the patient for 3 months after surgery.

**Outcomes:**

No significant signs of recurrence or metastasis were detected during the time.

**Lessons:**

Liposarcoma is rarely found in breast tissue. Moreover, it is difficult to distinguish between lipomas and liposarcomas by assistant examination. In this case report, we sought to enhance the awareness and understanding of breast liposarcoma. Understanding the clinical and pathological characteristics of this rare disease will aid clinicians in better recognition and diagnosis in future clinical practice, as well as in exploring more comprehensive and precise treatment plans and prognosis evaluation systems.

## 1. Introduction

According to the World Health Organization, over 100 distinct subtypes of soft tissue tumors are classified based on their potential tissue origins. Among these, liposarcoma is the most prevalent soft tissue sarcoma, constituting approximately 20% of all soft tissue malignancies in adults. Typically, liposarcomas are located in the deep soft tissues of the limbs and retroperitoneum, while their occurrence within the breast tissue is exceedingly rare.^[1]^ This report details an unusual case of an atypical lipomatous tumor/well-differentiated liposarcoma occurring in the breast.

## 2. Case presentation

A 65-year-old woman was admitted to our hospital with an “asymptomatic movable mass in the left breast that had persisted for approximately half a month.” Clinical examination revealed that the patient was obese (BMI: 31.56 kg/m²), with a palpable soft mass located in the inner upper quadrant of the left breast. The mass, measuring approximately 7 cm × 7 cm, was smooth, non-tender, and relatively well circumscribed. No significant mass was found in the right breast and no palpable lymphadenopathy was detected in the bilateral axillary or supraclavicular regions.

**Figure 1.**
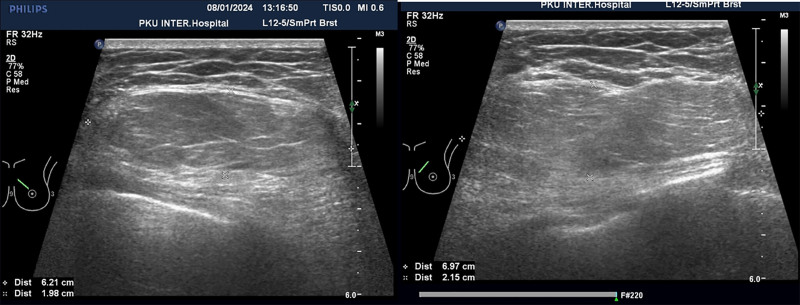
Ultrasound examination showed a large mass in the left breast.

**Figure 2.**
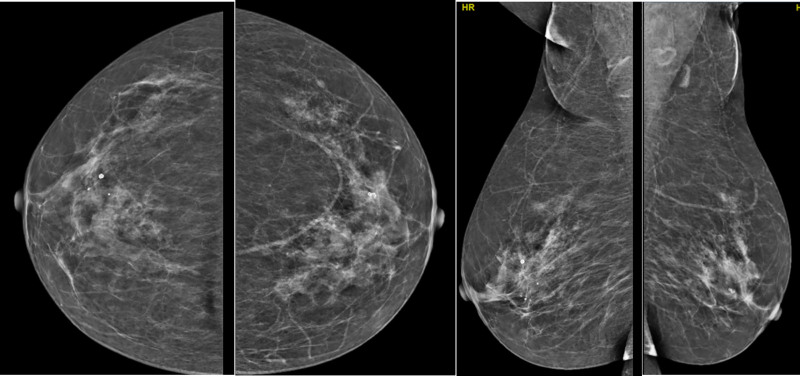
Mammography revealed scattered punctate calcifications in both breasts.

Preoperative ultrasound examination identified an isoechoic mass within the fatty layer of the left breast, measuring approximately 7.0 cm × 6.2 cm × 2.2 cm. The mass exhibited a regular shape but with unclear borders (Fig. 1). Color Doppler flow imaging did not reveal any significant blood flow within the mass, leading to a preliminary diagnosis of lipoma. Additionally, mammography revealed scattered punctate calcifications in both breasts without a clear mass, which was categorized as BI-RADS 2 (Fig. 2). The remaining preoperative laboratory tests and imaging studies showed no significant abnormalities or contraindications to surgery. Consequently, the patient underwent mass resection of the left breast under local anesthesia.

**Figure 3.**
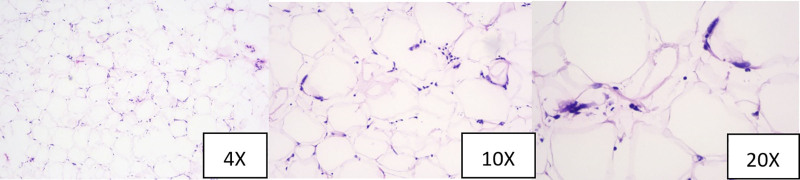
Pathological results at different magnifications.

**Figure 4.**
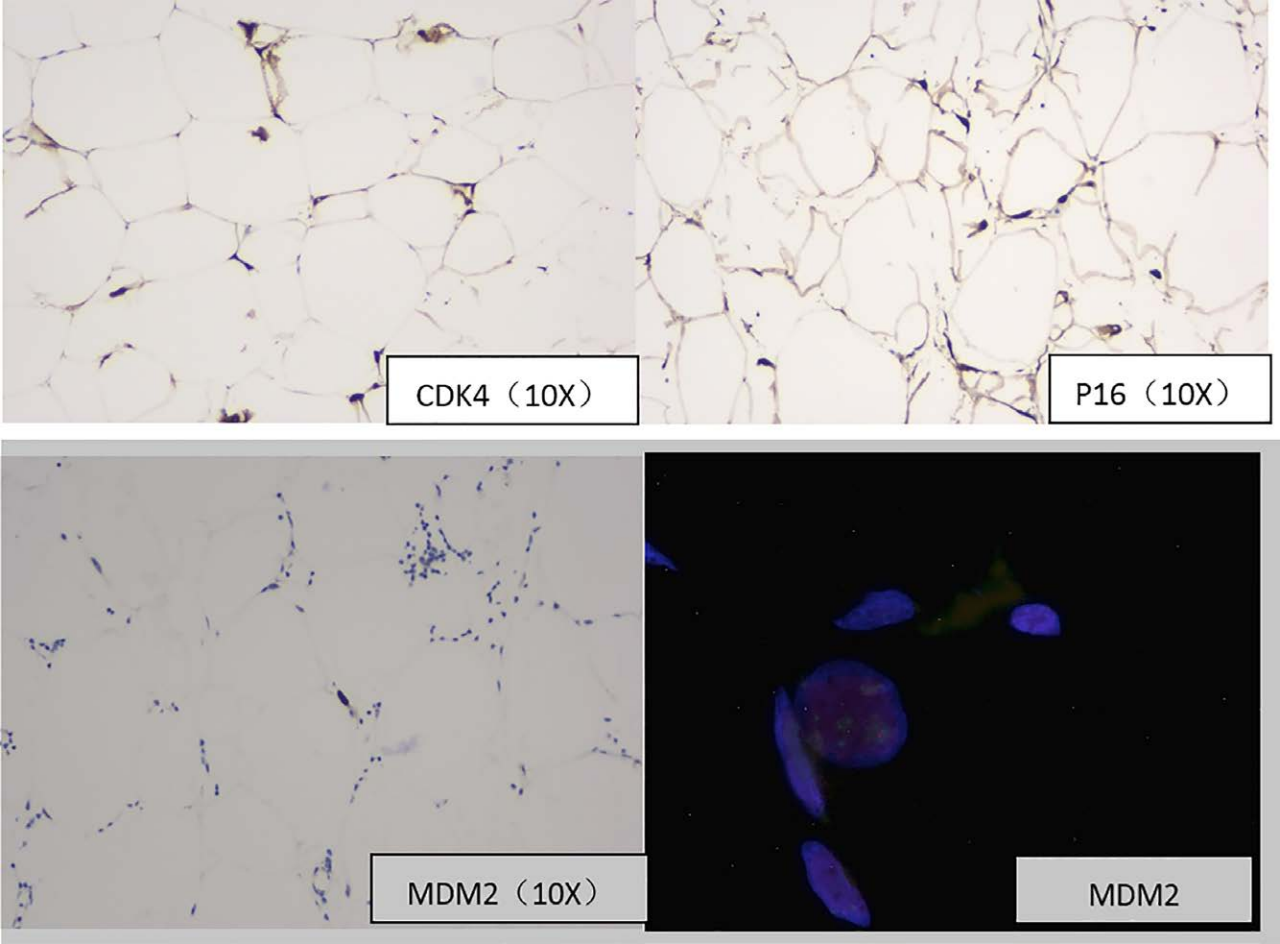
Pathological results of CDK4, P16, and MDM2.

Postoperative pathological analysis indicated that the mass in the left breast was a lipomatous tumor, characterized by the presence of a few enlarged cell nuclei and lymphocytic infiltration within the stroma (Fig. 3). The total tissue volume measured approximately 6 cm × 4.5 cm × 2.5 cm. Immunohistochemical assessments showed positivity for CDK 4 and P16, with minimal positivity for MDM 2. Furthermore, fluorescence in situ hybridization (FISH) analysis demonstrated flame-like amplification of the MDM 2 gene. The integration of FISH results with immunohistochemistry findings supported the diagnosis of atypical lipomatous tumor/well-differentiated liposarcoma (Fig. 4).

**Figure 5.**
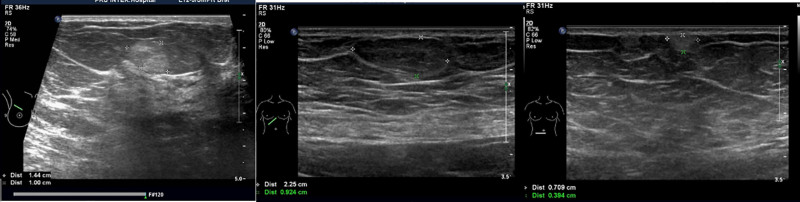
Ultrasound examination showed masses of other parts of the body.

In conjunction with the patient’s pathological findings, comprehensive chest, abdominal, and pelvic computed tomography examinations revealed no evidence of visceral metastasis. Notably, ultrasound examination indicated the presence of “multiple hyperechoic masses in the fatty layer of the right breast and multiple isoechoic and hyperechoic masses in the fatty layer of the right abdominal wall (as shown in Fig. 5).” Consequently, extended resection of the left breast lesion was performed along with excision of the right breast mass and mass in the right abdominal wall. Postoperative pathological analysis indicated clear resection margins for the left breast lesion, identified the right breast tumor as a hamartoma, and suggested that the mass on the right abdominal wall was lipoma.

## 3. Discussion

As the most prevalent type of adult soft tissue sarcoma, liposarcoma typically presents in the extremities and retroperitoneum. The fifth edition of the World Health Organization classification of soft tissue tumors categorizes liposarcomas into several types: well-differentiated liposarcoma, dedifferentiated liposarcoma, myxoid liposarcoma, pleomorphic liposarcoma, and myxoid pleomorphic liposarcoma.^[1]^ In contrast to retroperitoneal liposarcoma, certain well-differentiated liposarcomas occurring in the limbs and trunk are identified as “atypical lipomatous tumors,” as they can often be surgically excised with a negligible risk of metastasis.^[2]^

Liposarcoma predominantly affects individuals aged 50 to 70 years, with a male-to-female incidence ratio of approximately 4:1. Clinically, most liposarcomas present as slowly enlarging, painless masses that are often discovered incidentally during physical examinations. As the disease progresses, patients may experience symptoms, such as increased skin temperature, pain, and limited mobility. Liposarcomas that arise in deep tissues, such as the retroperitoneum or abdominal cavity, tend to remain asymptomatic in the early stages but may grow sufficiently large in later stages to compress adjacent organs, including the bladder, intestines, and kidneys, thereby causing corresponding symptoms.

Preoperative imaging is crucial for determining the origin and extent of the mass as well as for assessing metastasis. Ultrasound is commonly employed as an initial examination because it provides preliminary information regarding the type and location of the mass. However, this may be insufficient to distinguish between benign lesions (such as lipomas) and liposarcomas. In our case, which involved a liposarcoma located in the breast, a relatively rare occurrence, we selected ultrasound for the initial evaluation. For further imaging, if the mass is situated in the limbs, trunk, or head and neck, magnetic resonance imaging is preferred because of its ability to clearly delineate the anatomical relationships between the mass and the surrounding normal tissues. Conversely, if a mass is found in the retroperitoneum or abdominal cavity, computed tomography is recommended.^[3]^

Under scopic pathology classifies atypical lipomatous tumors/well-differentiated liposarcomas into 3 subtypes: adipocytic (lipoma-like), sclerosing, and inflammatory. The adipocytic subtype is the most prevalent, characterized by varying sizes of adipocytes interspersed with fibrous tissue and exhibiting trachychromatic nuclei within the lobules, fibrous septa, and walls of large blood vessels. The sclerosing subtype is distinguished by abundant fibrous elements and irregular cells that also display trachychromatic nuclei. The inflammatory subtype primarily consists of lymphocytes, with the stroma potentially containing aberrant cells and/or lipoblasts.^[1]^

Genetically, atypical lipomatous tumors/well-differentiated liposarcomas commonly exhibit amplification of the 12q13-q15 region, which encompasses the MDM2 and CDK4 genes. Consequently, FISH is frequently used to assess MDM2 amplification. A definitive diagnosis typically requires integration of histopathological morphology, immunohistochemical expression, and molecular genetic characteristics. Moreover, molecular and cytogenetic studies of various liposarcoma types are advancing, focusing on markers, such as FUS-DDIT3, tyrosine kinase receptors, PPAR-γ, and XPO1. These investigations have identified several markers associated with tumor development, thereby offering clinical insights and guidance for decision-making regarding the identification and treatment of different liposarcoma subtypes.^[4]^

The treatment of liposarcoma varies according to subtype. Surgical resection was the primary approach. Well-differentiated and dedifferentiated liposarcomas typically do not require neoadjuvant or adjuvant chemotherapies. Most well-differentiated liposarcomas, except those located in deep tissues such as the retroperitoneum, can often be treated surgically. The surgical principle is to achieve complete resection (R0) of the primary tumor without compromising the tumor margin. For lesions in which R0 resection is not feasible (R1 or R2), adjuvant radiotherapy may be used. Approximately 10% of well-differentiated liposarcomas can progress to dedifferentiated liposarcomas, which possess metastatic capabilities and are associated with significantly higher rates of local recurrence and poorer patient outcomes.^[5]^ Consequently, patients with palindromic or metastatic well-differentiated or dedifferentiated liposarcomas are advised to consider combined chemotherapy or participation in clinical trials. Neoadjuvant or adjuvant chemotherapy is also considered for myxoid liposarcoma and metastatic pleomorphic liposarcoma, although the utility of such treatments for pleomorphic liposarcoma remains debated.^[6]^ Given that most recurrences and metastases of soft tissue sarcomas typically occur within 2 years following treatment, some scholars advocate close monitoring of soft tissue sarcomas located in the limbs and superficial trunk during this period.^[7]^

The prognosis of patients with soft tissue sarcoma is influenced not only by the pathological type, but also by a range of factors. First, age and tumor location play significant roles; younger patients and those with tumors in the limbs exhibit a higher risk of recurrence, whereas patients with tumors in internal organs are more likely to succumb to systemic dissemination. Second, histological grade is an important factor, although the tumor site and histological specificity can limit prognostic predictions. Third, tumor size has been shown to correlate positively with the likelihood of local recurrence and distant metastasis; the larger the tumor, the higher is the probability of these complications. In addition, achieving of R0 resection during surgery is crucial for good prognosis.^[8]^ There are 2 widely used methods for predicting prognosis. The first is the Memorial Sloan Kettering Cancer Center sarcoma nomogram developed in 2002, which incorporates age, tumor size, histological grade, pathological subtype, and anatomical site. However, this nomogram primarily focuses on postoperative evaluation, limiting its applicability to preoperative assessment. The second method is the AJCC staging system, which evaluates anatomical site, tumor size, lymph node involvement, distant metastasis, and histological grade. Although it does not target specific pathological types, this system provides a standardized, internationally recognized framework for prognostic assessment. With advancements in molecular diagnostics, tumor markers, genomics, and related studies, prognostic evaluation systems are expected to improve significantly in the future.^[9]^

## 4. Conclusion

In our study, we examined an elderly woman with a breast tumor measuring >5 cm. The patient had no family history of the tumor. Radical surgical resection (R0 resection) was performed without subsequent radiotherapy or chemotherapy. We followed up the patient for 3 months through ultrasound examinations, during which no significant signs of recurrence or metastasis were detected. We recommend scheduling breast ultrasound for the patient every 6 months and breast mammography annually as part of the follow-up plan. This study had several limitations. First, while we referenced relevant guidelines and literature on soft tissue tumors, our treatment decision may not represent the optimal approach because there are limited reports and high-quality evidence available, along with a lack of accurate and individualized diagnostic and treatment plans. Second, despite conducting comprehensive preoperative examinations, challenges in differential diagnosis remain, indicating that clinicians should continue to enhance imaging techniques for early identification and diagnosis of malignant tumors in future practice. When indicated, we may initiate multidisciplinary team consultations with radiologists and pathologists, systematically collate and analyze cases, and summarize insights derived from these cases to develop evidence-based recommendations for future clinical practice. Lastly, the follow-up period for this patient was relatively short, necessitating regular monitoring to maintain vigilance regarding the risks of local recurrence and distant metastasis. In addition, there is a need to explore and develop a more comprehensive prognostic evaluation system.

## Acknowledgments

We would like to express our gratitude to the patient for granting permission to use their clinical data in this study and for the publication of this research.

## Author contributions

**Resources:** Xinrong Qu.

**Supervision:** Chun Zhang.

**Validation:** Xinrong Qu, Chun Zhang.

**Visualization:** Chun Zhang.

**Writing – original draft:** Xinrong Qu, Chen Li.

**Writing – review & editing:** Xinrong Qu.
